# Editorial: Emotional and social value of organizations

**DOI:** 10.3389/fpsyg.2022.1064540

**Published:** 2022-12-02

**Authors:** Virginia Barba-Sánchez, José Luis Retolaza, Leire San-José, Adrian Zicari

**Affiliations:** ^1^ENSITMA, Department of Business Administration, ESII, University of Castilla-La Mancha, Albacete, Spain; ^2^HUME, Deusto Business School, University of Deusto, Bilbao, Spain; ^3^ECRI, Financial Economics II, University of the Basque Country, Bilbao, Spain; ^4^Department of Accounting and Management Control, ESSEC Business School, Cergy, France

**Keywords:** social value, emotional value, stakeholder theory, shared value, social economy, social accounting

## Introduction

Financial and economic indicators have traditionally been used to measure organizational value. In recent years, however, there has been increasing interest in corporate social responsibility and in integrating both economic and social responsibilities into organizational value. The fostering of the social and solidarity economy (SSE) (Calderón-Milán et al., 2020), the economy based on the common good (Sanchis et al., 2021) and sustainability has exposed the shortcomings of this traditional methodology when it comes to calculating the organizational social value. Due to the fact that financial-economic indicators are still recognized as the main means of determining value, there is often a lack of integration between economic and social aspects, and while there are well-established measurements for corporate economic status, there is currently no standardized method for measuring organizational social value. Although previous initiatives such as the Global Reporting Initiative (GRI) or the Social Return on Investment (SROI) have attempted to go further than merely financial aspects, these do not permit a full comparison between companies and do not efficiently analyse corporate social return (Retolaza et al., 2015). A new business narrative is, therefore, needed to incorporate this social value (Retolaza Ávalos et al., 2021).

A number of Research Topics are worthy of mention. In 2011, the authors Retolaza and San-José published the paper “Social Economy and Stakeholder Theory, an Integrative Framework for the Socialization of Capitalism” in CIRIEC-España, in which they outlined the basis of their future model. In 2016, San-José and Retolaza in collaboration with Springer published a social accounting manual for stakeholders which systematized the process to monetise the total social value generated by an organization and broke it down for each stakeholder. In 2020, a special issue entitled “Social Accounting: Monetising the Social Impact of Companies and Organizations” was published in CIRIEC-España, and in 2021, another special issue entitled “Applying Social Value to Organizations” was also published in the Bulletin of Economic Studies. While previous studies have improved the understanding of the social value of organizations, gaps remain and further research is needed given the complexity and extent that such calculation requires. One clear example of these gaps are the attempts to quantify emotional value and these are extremely limited and incomplete. Affective events theory focuses on the role of emotion and the reaction to an event is based on the attainment of an individual's personal goals and values (Stylos et al., 2022). People are growing increasingly concerned about the impact of economic activity on the environment and society. In this context, although organizations seek to publicly legitimize their activity, accounting is often inadequate to identify the organizational distributed value for its different stakeholders in monetary terms (Retolaza Ávalos et al., 2021). Further research is, therefore, needed in order to understand the social value, in general, and the emotional value, in particular, which is generated by organizations, especially by identifying the social value perceived by the various stakeholders.

The aim of our research is to achieve an integrated way of representing organizational value to include both the generated value aspects and the financial ones. In particular, one of the most innovative aspects addressed in this special issue is that of emotional value. Given its computational complexity, emotional value has always been relegated to future research. This issue includes 15 papers which focus on various holistic paradigms, for example, the Social Accounting for Stakeholders or Stakeholder Accounting (SAS) model. Rather than focusing purely on factors relating to economic activity, we aim to explore the processes that engage stakeholders. Furthermore, contributors apply the idea of social value to a variety of settings: universities, science and technology parks, disability organizations, food markets and the sports industry.

By applying the SAS model, we can enable a quantitative and monetised comparison of integrated value between companies. The inclusion of the emotional value would allow not only more efficient decision-making based on symmetry but also more comprehensive information (private organizations), more efficient consumption or investment decisions (private individuals) and efficient indicators for establishing public policies (public administration). This social value could well prove to be a basic, valuable corporate reputation component since it represents a way of visualizing their social contribution and their level of corporate social responsibility. The editorial team has, therefore, announced a call for research into innovative ways of measuring the organization's social value according to its social and economic contribution but without overlooking the generation of emotional value. The researchers have presented 15 original research contributions to this special issue to exemplify the contribution of different aspects including the financial aspect to the value generated by an organization.

## Economic and social value

The monetised social accounting model provides constant feedback. It is necessary to strengthen and consolidate the model with further case studies in order to validate and standardize its principles in different economic and social sectors and types of organizations. In this regard, this special issue includes a number of practical applications to enrich the existing knowledge base and which endorse model validity. In their paper, Torres-Pruñonosa, Raya et al. outline a system to measure the economic and social value of TecnoCampus, a Science and Technology Park in the city of Mataró in the Maresme region of Catalonia in Spain, which is based on the input-output model combined with the social accounting matrix. Areiza-Padilla and Puertas, meanwhile, analyse the case of Starbucks in Colombia as a global and sustainable brand that positively contributes to sustainability in emerging markets and is beneficial both for the environment and society in general. The authors Guzmán-Pérez et al. focus their analysis on the social value and urban sustainability of food markets using the integrated social value model and obtain new, relevant and understandable social sustainability indicators for stakeholders. In another article, Cearra et al. highlight the creation of social value through cooperation and co-creation in the context of an ecosystem supporting entrepreneurship based on a technological platform and an ad hoc methodology seeking the involvement and collaboration of all stakeholders. Finally, Gutiérrez-Goiria et al. apply the Global Reporting Initiative (GRI) model to measure the social value generated by European universities for their stakeholders and identify various examples of good practices that could be used to improve reporting standards in order to better reflect social value.

It is also important, however, to emphasize the progress that has already been made by authors such as Torres-Pruñonosa, Plaza-Navas et al. in their application of a bibliometric analysis which maps the intellectual structure of scholarship on economic and social value in the sports industry. Correspondingly, in a similar way to the recent analysis of other industries, both the creation of social value indicators for sports entities and the empirical analysis of social efficiency in sports institutions are identified and outlined as future areas of research. In turn, Pascucci et al. conduct a systematic review of the research that reconciles organizational profits and social functions. Using the PRISMA method, they find that certain records mention the socio-emotional value relating to organizational and employee suffering, while other articles consider this to be a positive factor that improves performance and prevents problems in organizations.

## Emotional value of organizations

The subject of the emotional value of organizations is covered in four papers which seek to quantify the emotional value generated by organizations. The authors Retolaza and San-José propose a mechanism to measure stakeholder satisfaction and its fair value using the Net Promoter Score (NPS), which the authors claim is a simpler tool for measuring satisfaction and loyalty than the earlier SERVQUAL model proposed by Ruiz-Roqueñi (2020). In turn, Ruiz-Roqueñi proposes an alternative way to monetise the emotional value dimension in this special issue through a correction factor which is applied to the value variables identified. More specifically, they also propose that reputation be considered as a correction factor, given its impact on the link between financial/economic performance and social performance. Additionally, their findings show that most of the emotional value created in a social organization arises from its interactions with its members.

In their paper, Hong et al. introduce the positive effect of emotions in the sphere of the information and communication technology industry by relating it with teleworking during the COVID-19 pandemic, while Zhang et al. consider the specific aspects of emotional regulation in Chinese culture, otherwise known as guanxi harmony. In both cases, the results suggest that if human resource managers wish to enhance workers' remote engagement or promote employee motivation, they should apply appropriate emotion regulation strategies.

The psycho-emotional aspect of social value creation is also analyzed in the paper by Margaça et al. based on establishing social companies aligned with the 2030 Agenda to achieve the Sustainable Development Goals (SDGs). By comparing Portugal and Spain using the dimensions of the Theory of Planned Behavior (TPB), they measure spirituality and optimism as sources of emotional value in the process to create a company in the social economy.

## Social value of disability organizations

Finally, three papers focus on disability organizations as social value generators. The first paper by Ortiz García and Olaz Capitán researches value generation by entrepreneurship for people with disabilities (PwDs), a particularly vulnerable group with high rates of unemployment, and this is based on a sample of 224 entrepreneurs with disabilities (EwDs). In the second paper, Barba-Sánchez et al. explore the subject in greater depth by analyzing a specific case of entrepreneurship by people with intellectual disabilities (PwIDs), Abono Café, and how it has contributed to improving their job integration and their network of social relations by generating social value in their immediate surroundings, and this confirms the results presented by Barba-Sánchez et al. (2021). In the third paper, Tirado-Valencia et al., examine how third-sector organizations working in the disability sector account for the emotional value they create for their stakeholders. These disability organizations, which are either created by EwDs or which hire PwDs, generate social value not only for this vulnerable group of people but also for all other stakeholders, given that employment contributes to the psycho-social and economic development of individuals while increasing the social well-being of the community (Wiklund et al., 2019).

## Author contributions

All authors listed have made a substantial, direct, and intellectual contribution to the work and approved it for publication.

## Funding

This study was supported by the University of the Basque Country under Grant US20/11, the Consejería de Educación, Cultura y Deportes (JCCM), and the European Regional Development Fund (ERDF) under Grant SBPLY/21/180501/000192.

## Conflict of interest

The authors declare that the research was conducted in the absence of any commercial or financial relationships that could be construed as a potential conflict of interest.

## Publisher's note

All claims expressed in this article are solely those of the authors and do not necessarily represent those of their affiliated organizations, or those of the publisher, the editors and the reviewers. Any product that may be evaluated in this article, or claim that may be made by its manufacturer, is not guaranteed or endorsed by the publisher.
